# Coping strategies and correlations with depressive symptoms among female nurses working in Japanese general hospitals

**DOI:** 10.3389/fpubh.2024.1422395

**Published:** 2025-01-14

**Authors:** Hideyuki Kubo, Yoshiyuki Kaneko, Kaori Saitoh, Ryuji Furihata, Maki Jike, Yuichiro Otsuka, Makoto Uchiyama, Masahiro Suzuki

**Affiliations:** ^1^Department of Psychiatry, Nihon University School of Medicine, Tokyo, Japan; ^2^Fondation FondaMental, Hôpital Albert Chenevier Pôle de Psychiatrie, Créteil, France; ^3^Agency for Student Support and Disability Resources, Kyoto University, Kyoto, Japan; ^4^Department of Food Safety and Management, Faculty of Food and Health Sciences, Showa Women's University, Tokyo, Japan; ^5^Division of Public Health, Department of Social Medicine, Nihon University School of Medicine, Tokyo, Japan; ^6^Tokyo Adachi Hospital, Tokyo, Japan

**Keywords:** depression, healthcare worker, nurse, occupational health, stress coping

## Abstract

**Introduction:**

Preventing depression among nurses is a critical issue from the perspective of occupational welfare, but associations between depressive symptoms in nurses and stress-coping strategies remain unclear.

**Methods:**

In the present study, an epidemiological study was conducted based on a cross-sectional questionnaire survey. Data obtained from 2,534 female nurses working at three general hospitals in Tokyo, Japan, were analyzed. Participants completed a questionnaire comprising 42 items, including depressive symptoms and stress-coping strategies, in addition to sociodemographic information and perceived mental stress.

**Results:**

Our study showed that the emotional distraction strategy “Engaging in hobbies or relaxing” was negatively associated with both depressed mood and loss of interest. In addition, the problem-solving strategy “Making an effort to think optimistically” was negatively associated with loss of interest. Conversely, use of avoidant strategies was positively associated with both depressive symptoms.

**Discussion:**

Our findings may indicate the importance of focusing on types of coping styles when developing strategies to prevent depressive symptoms in nurses.

## 1 Introduction

In recent years, the mental wellbeing of healthcare professionals has become an important issue from the perspective of occupational welfare. Among the various healthcare-related professions, nurses are the most likely to experience a variety of occupational stressors (1), including frequent decision-making, heavy workload, irregular work schedule, and difficult patients and their families, making mental health measures for nurses a priority issue. Among the many factors related to the mental health of nurses, depression is a particularly common problem related to continued work (2, 3). Recently, the relationship between stress and depression has been becoming clearer in studies involving nurses (4, 5), in addition to a general population study (6). Development of appropriate measures to cope with stress is expected to aid in the prevention of depression.

Stress coping is the process by which cognitive or behavioral strategies work to adapt to or tolerate a specific source of psychological stress (7). The relationships between coping strategies and health outcomes have been examined extensively, primarily on the basis of classification into the three categories of problem-solving, emotion-focused, and avoidant strategies (8, 9). A problem-solving strategy is one in which action is taken to change the situation causing stress. An emotional arousal strategy is one in which action is taken to change the individual’s own experience of the negative emotions that cause stress. An avoidant strategy is one in which the individual strives to distance themself from the cause of the stress. These different strategies are known to have differing effects on many health outcomes (9, 10). More specifically, several studies conducted in clinical settings (11, 12) and an epidemiological setting (8, 13) have reported that problem-focused coping strategies are associated with a decreased risk of depression, whereas some emotion-focused coping strategies and avoidant strategies are associated with increased risks of depression. Furthermore, stress-coping strategies are beginning to be examined by occupation, including nursing (14, 15), based on the idea that factors and risks will vary by occupation. The associations between the coping strategies used by nurses and depressive symptoms may differ from those in the general population, since nurses, especially hospital nurses, are more susceptible to a range of stressors such as workload, uncertain treatment, conflict with doctors and other nurses, and death and dying (16), and they may experience difficulty finding sufficient time to cope with stress. However, to the best of our knowledge, no studies have examined the relationship between depression and stress-coping strategies in hospital nurses, beyond a small number of relatively small-scale studies (17, 18). Identifying the relationship between depression and stress-coping strategies in hospital nurses may be meaningful in view of previous findings that depression and suicidal ideation are associated with workplace stress (19, 20).

An epidemiological survey was conducted among three samples of hospital nurses to identify stress-coping strategies associated with depressive symptoms. We investigated whether any correlations exist between the two core depressive symptoms of “depressed mood” and “loss of interest” and stress-coping strategies among more than 3,000 female nurses working in Japanese general hospitals. The research question in the present study was: which strategies are positively or negatively associated with depressive symptoms in hospital nurses?

## 2 Materials and methods

### 2.1 Participants

We administered a cross-sectional questionnaire-based epidemiological survey of sleep, mental health, and lifestyle to nurses in 2015 (21). In this study, data from a multicenter survey were collected to avoid bias in the data due to hospital characteristics. Participants were nursing staff working at general hospitals in Tokyo, Japan. Three general hospitals (Hospitals A, B, and C) affiliated with different medical schools and each having about 1,000–1,100 beds participated in the present survey. The Director of Nursing at each institution explained the purpose of the present study and requested the cooperation of the heads of wards or outpatient divisions of all staff working at each hospital. The heads provided each of their subordinate nursing staff with an envelope containing information about the purpose of the survey, a request to participate, a questionnaire on sleep, mental health, and lifestyle, and an envelope for returning the completed questionnaire. Participation in the survey was initiated by returning the sealed envelope to the Director of Nursing, which ensured anonymity. The envelopes were grouped by the Director of Nursing and delivered to the administrative staff. As for the nursing staff who did not want to participate in this study, each staff member was also required to provide a letter stating that he/she did not wish to participate, which was returned to staff outside the hospital organization via the head of nursing in a sealed envelope. It was indicated in writing that their privacy was protected and that they would not be disadvantaged if they did not cooperate with this survey, and only asked them to complete the survey if they gave their consent. A total of 3,037 nurses (Hospital A, *n* = 1,042; Hospital B, *n* = 980; Hospital C, *n* = 1,015) from the three hospitals were selected for the survey. The total number of participants was 2,692, for a collection rate of 88.6%. Among these 2,692 participants, those for whom data on sex (*n* = 12), age (*n* = 15), or self-reported depressive symptoms (*n* = 6) were missing were excluded from the analysis. Given the small numbers, male nurses (*n* = 125) were also not included. We analyzed data from the final sample of 2,534 female nurses. The mean age of the participants was 31.1 ± 9.0 years. Demographic characteristics of participants are presented in Table 1. All study protocols were approved by the Ethics Committee of Nihon University School of Medicine (approval number: 27–1-0).

**Table 1:** Demographic characteristics for each of the age groups.

				Age			
		20–29		30–39		40–	
	n	n	%	n	%	n	%
Total	2,534	1,381	54.5	696	27.5	457	18.0
Department							
Medical	1,160	690	59.5	313	27.0	157	13.5
Surgical	665	379	57.0	165	24.8	121	18.2
Others	709	312	44.0	218	30.7	179	25.2
Shift work							
Once or more per month	2016	1,260	62.5	495	24.6	261	12.9
Less than once per month	515	119	23.1	201	39.0	195	37.9
Professional position							
Chief	338	21	6.2	140	41.4	177	52.4
Staff	2,181	1,350	61.9	554	25.4	277	12.7
Psychological stress							
Stressed	2027	1,089	53.7	559	27.6	379	18.7
Unstressed	507	292	57.6	137	27.0	78	15.4

### 2.2 Procedures

For this survey, we developed a self-administered questionnaire, which asked about sociodemographic characteristics and contained 42 individual questions focusing on sleep, mental health, and lifestyle. The 42 items were categorized into: (1) sociodemographic information such as age, hospital, department, position, and night shift; (2) perceived mental stress; (3) depressive symptoms (depressed mood and loss of interest); and (4) stress-coping strategies.

Sociodemographic variables included age (20–29 years, 30–39 years, and ≥ 40 years), hospital (A, B or C), department in the hospital (internal medicine, surgery or other), professional position (head or staff). Present engagement in night shifts was examined by asking the frequency (less than once per month or once or more per month).

Perceived mental stress was evaluated by asking “Was there stress due to dissatisfaction, worries or hardship?” This question included the following response items: “much,” “some,” “little” or “none.” Respondents answering “much” or “some” were defined as stressed, whereas those answering “little” or “none” were defined as unstressed.

Two symptoms for depression during the previous month were included in the questionnaire (21, 22):

(1) Have you been bothered by feeling down, depressed, or hopeless? (“never”/“seldom”/“sometimes”/“often”): depressed mood.

(2) Have you been bothered by little interest or pleasure in doing things than usual? (“never”/“seldom”/“sometimes”/“often”): loss of interest.

Participants who answered “often” to Question 1 or 2 were classified as having a depressed mood or loss of interest, respectively (21, 22). Cronbach’s alpha, which represents internal consistency, was 0.56 in the present study, lower than that in the previous study of postpartum depression (23). In this study, the area under the curve reflecting validity was 0.84.

To explore the stress-coping strategies of participants, the following 12 questions taken from questionnaires used in prior epidemiological surveys conducted by the Japanese Ministry of Health, Labour and Welfare (13) were embedded in the questionnaire. These 12 items differentiated among three different coping strategies: problem-focused (1 and 2); emotion-focused (3–10); and avoidant (11 and 12). Participants were asked to answer the question “Do you use the following coping strategy when you have stress?” for: (1) Making an effort to solve problems actively (yes/no); (2) Making an effort to think optimistically (yes/no); (3) Venting emotions by talking to others (yes/no); (4) Engaging in hobbies or relaxing (yes/no); (5) Engaging in sports (yes/no); (6) Watching TV/listening to the radio (yes/no); (7) Eating (yes/no); (8) Drinking alcoholic beverages (yes/no); (9) Smoking (yes/no); (10) Seeking strong stimuli or excitement (yes/no); (11) Giving up attempts to solve problems (yes/no); and (12) Bearing stress without action (yes/no).

### 2.3 Statistical analyses

Differences in the prevalence of depressive symptoms were compared between groups with different sociodemographic characteristics (age, hospital, department, night shift, position) and perceived psychological stress using chi-squared tests. Differences in the percentage of respondents to stress-coping questionnaires were also compared between age groups using chi-squared tests. Logistic regression analysis was used to assess the associations of stress-coping strategies with the two core depressive symptoms. We initially examined all variables in univariate models. Multivariate logistic regression analyses were then performed to adjust for the confounding effects of sociodemographic factors (sex, age, hospital, department, position, and night shift), and perceived mental stress. We then performed additional multiple logistic regression analyses for all items that showed a significant association in multivariate models to assess for the confounding effects of other significant items, in addition to sociodemographic factors and perceived mental stress. Significance was set at the 5% level. All analyses were performed using SPSS version 24 software (IBM Corporation, Armonk, NY, United States).

## 3 Results

### 3.1 Prevalence of depressive symptoms

The prevalence of depressive symptoms by age group, hospital department, engagement in night shift, professional position, and psychological stress is presented in Table 2. Depressed mood and loss of interest were found in 14.9 and 8.5% of participants, respectively. The prevalence of depressed mood differed by age, frequency of night shift, and presence of psychological stress. The prevalence of loss of interest differed by age and presence of stress. Perceived stress was seen to be associated with increased prevalences of both core depressive symptoms.

**Table 2:** Prevalence of depressive symptoms for each of the groups defined by sociodemographic factors.

		Depressed mood		Loss of interest	
	n	n	%	n	%
Total		377	14.9	215	8.5
Age, y					
20–29	1,381	218	15.8	131	9.5
30–39	696	82	11.8	44	6.3
≥40	457	77	16.8	40	8.8
Chi-squared			7.6		6.0
*p*-value			0.023		0.049
Department					
Medical	1,160	175	15.1	101	8.7
Surgical	665	103	15.5	57	8.6
Other	709	99	13.9	57	8.0
Chi-squared			0.7		0.3
*p*-value			ns		ns
Professional position					
Head	338	51	15.1	24	7.1
Staff	2,181	323	14.8	191	8.8
Chi-squared			0.0		1.0
*p*-value			ns		ns
Night shift					
Once or more per month	2016	318	15.8	181	9.0
Less than once per month	515	59	11.4	34	6.6
Chi-squared			6.1		3
*p*-value			0.013		ns
Perceived mental stress					
Stressed	2027	366	18.0	209	10.3
Unstressed	507	11	2.2	6	1.2
Chi-squared			80.6		43.4
*p*-value			< 0.001		< 0.001

ns, not significant.

### 3.2 Types of coping strategy

The stress-coping strategies incorporated in the questionnaire and their prevalence by age group are summarized in Table 3. One item included in problem-solving strategies and one in avoidant strategies showed effects of age. Effects of age were found for all items in emotion-focused strategies. The predominance of younger age groups was apparent for several items in emotion-focused strategies.

**Table 3:** Percentage of respondents for stress-coping questionnaires by age group.

	Respondents (%)					
	Total	Age (y)				
Stress-coping strategy		20–29	30–39	≥40	Chi-squared	*p*-value
Problem-solving strategy						
Making an effort to solve problems actively	15.4	13.5	18.5	16.6	9.6	0.008
Making an effort to think optimistically	36.3	37.3	32.7	38.7	5.7	ns
Emotional distraction strategy						
Venting emotions by talking to others	65.7	71.4	64.3	50.8	65.9	<0.001
Engaging in hobbies or relaxing	61.1	69.2	54.6	46.8	89.6	<0.001
Engaging in sports	19.5	19.8	21.5	15.5	6.4	0.040
Watching TV/listening to the radio	36.9	40.0	31.9	35.2	13.7	<0.001
Eating	41.9	47.0	40.7	28.0	51.6	<0.001
Drinking alcoholic beverages	31.5	34.2	31.2	23.4	18.6	<0.001
Smoking	6.6	5.9	6.4	9.2	6.2	0.045
Seeking strong stimuli or excitement	4.3	6.2	2.7	0.7	31.7	<0.001
Avoidant strategy						
Giving up attempts to solve problems	8.8	9.3	9.6	5.7	6.6	0.037
Bearing stress without action	27.2	27.2	25.8	29.1	1.5	ns

ns, not significant.

### 3.3 Association between stress-coping strategies and depressive symptoms

After adjusting for the confounding effects of age, hospital, department, professional position, night shift, perceived mental stress, and other stress-coping strategies, multivariate logistic regression analyses showed several types of associations between depressed mood and coping strategies (Table 4). Positive associations were found for the two avoidant strategies (“Giving up attempts to solve problems” and “Bearing stress without action”), whereas negative associations were found for “Engaging in hobbies or relaxing” as an emotion-focused strategy.

**Table 4:** Association between stress-coping strategies and depressed mood.

	Crude			Adjusted 1^1^			Adjusted 2^2^		
	OR	95%CI	*p*-value	OR	95%CI	*p*-value	OR	95%CI	*p*-value
Problem-solving strategy									
Making an effort to solve problems actively	0.79	0.58–1.09	ns						
Making an effort to think optimistically	0.84	0.67–1.06	ns						
Emotional distraction strategy									
Venting emotions by talking to others	1.06	0.84–1.33	ns						
Engaging in hobbies or relaxing	0.72*	0.58–0.90	0.004	0.73*	0.58–0.92	0.007	0.73*	0.58–0.92	0.007
Engaging in sports	0.81	0.60–1.08	ns						
Watching TV/listening to the radio	0.87	0.69–1.10	ns						
Eating	1.23	0.99–1.53	ns						
Drinking alcoholic beverages	1.15	0.91–1.45	ns						
Smoking	1.10	0.72–1.69	ns						
Seeking strong stimuli or excitement	1.23	0.74–2.05	ns						
Avoidant strategy									
Giving up attempts to solve problems	1.82*	1.30–2.54	<0.001	1.65*	1.17–2.31	0.004	1.56*	1.10–2.19	0.012
Bearing stress without action	1.91*	1.52–2.40	<0.001	1.61*	1.27–2.04	<0.001	1.76*	1.39–2.23	<0.001

ns, not significant; *p-value < 0.05.

1 Adjusted by age group, hospital, department in the hospital, professional position, night shift, and perceived mental stress.

2 Adjusted by age group, hospital, department in the hospital, professional position, night shift, perceived mental stress, and stress-coping strategies showing significant associations with depressed mood in Adjusted 1.

The final multivariate logistic regression analysis showed that problem-solving (“Making an effort to think optimistically”) and emotion-focused (“Engaging in hobbies or relaxing”) strategies were negatively associated with loss of interest (Table 5). The two avoidant strategies (“Giving up attempts to solve problems” and “Bearing stress without action”) were positively associated with loss of interest.

**Table 5:** Associations between stress-coping strategies and loss of interest.

	Crude			Adjusted 1^1^			Adjusted 2^2^		
	OR	95%CI	*p*-value	OR	95%CI	*p*-value	OR	95%CI	*p*-value
Problem-solving strategy									
Making an effort to solve problems actively	0.64*	0.41–0.99	0.046	0.69	0.44–1.08	ns			
Making an effort to think optimistically	0.64*	0.47–0.87	0.005	0.64*	0.47–0.88	0.006	0.61*	0.45–0.84	0.002
Emotional distraction strategy									
Venting emotions by talking to others	0.89	0.67–1.19	ns						
Engaging in hobbies or relaxing	0.53*	0.40–0.71	<0.001	0.51*	0.38–0.69	<0.001	0.53*	0.39–0.70	<0.001
Engaging in sports	0.79	0.54–1.15	ns						
Watching TV/listening to the radio	0.89	0.66–1.19	ns						
Eating	0.99	0.75–1.33	ns						
Drinking alcoholic beverages	1.06	0.79–1.43	ns						
Smoking	1.50	0.92–2.45	ns						
Seeking strong stimuli or excitement	1.65	0.92–2.94	ns						
Avoidant strategy									
Giving up attempts to solve problems	1.88*	1.25–2.82	0.003	1.68*	1.11–2.54	0.014	1.77*	1.16–2.72	0.009
Bearing stress without action	1.59*	1.19–2.13	0.002	1.38*	1.02–1.85	0.035	1.49*	1.10–2.02	0.010

ns, not significant; *p-value < 0.05.

1 Adjusted by age group, hospital, department in the hospital, professional position, night shift, and perceived mental stress.

2 Adjusted by age group, hospital, department in the hospital, professional position, night shift, perceived mental stress, and stress-coping strategies showing significant associations with loss of interest in Adjusted 1.

## 4 Discussion

In the present study, we surveyed depressive symptoms and stress-coping strategies among female nurses working in three general hospitals affiliated with different medical schools by asking participants to choose the applied coping strategies from among 2 problem-solving, 8 emotion-focused and 2 avoidant strategies. We identified the following associations between stress-coping strategies and depressive symptoms: (1) some problem-solving and emotion-focused strategies showed negative associations with depressive symptoms, but none showed any positive associations with depressive symptoms; (2) avoidant coping strategies exclusively showed positive associations with depressive symptoms; and (3) the number of coping strategies showing significant associations differed between the two core depressive symptoms.

### 4.1 Prevalence of depressive symptoms

The prevalence of each depressive symptom in our study was 8.5–14.9%, comparable to that in women from a previous general population study (24). The prevalence of depressed mood was highest in participants ≥40 years old, where administrative tasks are often required, whereas loss of interest was highest in 20–29 year olds, who are just starting nursing and need to become accustomed to their career.

### 4.2 Associations between coping strategies and age

Many emotion-focused strategies showed higher prevalences in younger age groups, in accordance with a previous general population study from our group (13). In contrast, no such tendency was found for problem-solving or avoidant strategies. The reason only emotion-focused strategies correlated with age is unclear, but this could be interpreted as meaning that strategies such as engaging in sports or talking to others may be preferred by younger generations.

Compared with women in our previous general population study (13), the strategies used much more frequently by nurses in our study (percentage difference > 10%) were “Venting emotions by talking to others,” “Engaging in hobbies or relaxing,” “Eating,” “Drinking alcoholic beverages,” and “Bearing stress without action.” However, the only strategy that nurses used much less frequently (percentage difference > 10%) than women from the general population sample (13) was “Smoking.” This suggests that nurses tend to use emotional distraction strategies other than smoking. This may be an indication that nurses are more aware of stress-coping strategies due to their occupational characteristics. Moreover, a higher awareness of the benefits of avoiding smoking as healthcare professionals may have been related to this difference.

### 4.3 Association between stress-coping strategies and depressive symptoms

Our study showed that the emotional distraction strategy of “Engaging in hobbies or relaxing” was negatively associated with both core depressive symptoms. This result was in line with a previous study showing that the coping style of “Taking his/her ease,” which belongs to the emotional distraction strategy, was negatively associated with depression among females in the general population (13). Although the present study did not examine causal relationships, our result may suggest that taking one’s ease reduces depressive symptoms in nurses; previous meta-analysis and systematic review studies have shown relaxation as significantly reducing depressive symptoms (25, 26). Moreover, our results indicated that the problem-solving strategy “Making an effort to think optimistically” was negatively associated with loss of interest. Various studies using multivariate analyses have shown that negative thinking can predict future depression (27, 28). Given these findings, introducing the strategy of optimistic thinking can also be expected to help reduce depressive symptoms in nurses. Unlike a previous study of a general population (13), the problem-solving strategy “Making an effort to solve problems actively” did not show an association with any of the core depressive symptoms. This may be because problems in nursing are often difficult to handle or solve. Distracting themselves through hobbies or relaxation may thus be more effective in stress coping for nurses than directly aiming to solve problems. Further, providing nurses with sufficient time for hobbies and relaxation is considered important from the perspective of stress management.

Moreover, use of avoidant strategies was positively associated with both depressed mood and loss of interest. This result is consistent with many previous studies in both clinical (29, 30) and community (13) settings, which have shown that avoidant strategies were positively associated with depressive symptoms. In addition, longitudinal studies have indicated that avoidant strategies are predictive of future depression (31, 32) and have suggested that life stressors may mediate the association between baseline avoidant strategies and risk of depressive symptoms (31). Our results, together with these preceding findings, suggest that use of avoidant strategies may be inadvisable for nurses, who generally tend to experience many stressors.

Combining the results of the present study with those of a recent cluster analysis of nurse data may help us better understand the appropriate nurse coping style to prevent or alleviate depressive symptoms (18). In this study, participants were classified into three groups according to the coping style used (emotional-response type, problem-solving type, and reality-escape type), and we compared the magnitude of K6 scores used to screen for depression and anxiety in each group. The results showed that the problem-solving type cluster had lower K6 scores than the other two clusters, suggesting the usefulness of the problem-solving strategy as well as our results. Combining these results with our findings above, together with another study that showed a negative relationship between taking a problem-solving strategy and psychological distress (33), it may be suggested that problem-solving strategies are the main methods to improve mental conditions including depressive symptoms, whereas taking time for hobbies and relaxation may also be a promising method. In the future, it would be desirable to conduct a longitudinal study to establish stress-coping strategies for nurses and to examine which stress-coping strategies may reduce or prevent depressive symptoms.

### 4.4 Limitations

The present study had several limitations that should be kept in mind when interpreting the results. First, because this was a cross-sectional study, causal relationships between stress-coping strategies and symptoms of depression could not be elucidated. The investigation of causality was beyond the scope of this study, so longitudinal research is needed to clarify such causal relationships. Second, because this study used a self-rating questionnaire to assess depressive symptoms, the lack of objectivity may have been an issue. Further improvements, such as the introduction of interview methods, are desirable in future work. Third, this study only targeted female nurses and did not examine the association between stress-coping strategies and depressive symptoms in male nurses, since most nurses in these hospitals were female at the time the data for this study were collected. By accumulating data from hospitals where many male nurses work, we would like to examine the association between stress-coping strategies and depressive symptoms in male nurses and examine the differences with women. Fourth, all data used in our study were from nurses working in hospitals in Tokyo, so it is unclear whether similar results would be obtained in hospitals in other countries or regions, given a report of cultural and regional differences influencing coping style. Future studies should clarify regional differences in coping strategies to reduce depressive symptoms. Finally, the present study did not assess psychiatric or physical comorbidities, which may have influenced the results.

## 5 Conclusion

The present study demonstrated that the prevalence of different depressive symptoms in nurses working at general hospitals in Japan was associated with different types of stress-coping strategies. Our findings may indicate the importance of focusing on certain types of coping styles, such as engaging in hobbies or relaxing, when developing strategies for preventing depression in nurses. Incorporating coping strategies into the training or continued education of nurses or as a leisure activity for nurses may be helpful to implement in hospital settings. Further longitudinal studies that examine the causal relationships between stress-coping strategies and symptoms of depression are needed.

## Data Availability

Derived data supporting the findings of this study are available from the corresponding author Yoshiyuki Kaneko on request (kaneko.yoshiyuki@nihon-u.ac.jp).
